# Remediation: Ground Zero for Uranium?

**DOI:** 10.1289/ehp.115-a298b

**Published:** 2007-06

**Authors:** Harvey Black

By harnessing bacteria that “breathe” uranium the way animals breathe oxygen, Florida State University microbiologist Joel Kostka is working to solidify liquid uranium waste under a 243-acre site adjacent to Oak Ridge National Laboratory. Using bacteria to solidify the waste *in situ* may be the best alternative to dealing with the waste, says Kostka. Other solutions, such as excavating and disposing of it elsewhere, are “just moving the problem, and [don’t] get rid of the waste,” says Kostka.

Kostka directs a team of microbiologists studying bioremediation as part of a five-year, $15-million U.S. DOE project led by Oak Ridge National Laboratory. The multi-disciplinary project includes geologists, geochemists, and microbiologists. and focuses on waste dumped underground over a 32-year span beginning in 1951. The waste is the result of the uranium enrichment process in weapons manufacturing. As much as 1 trillion liters of waste were dumped each year.

Kostka, whose work is described in the 1 February 2007 issue of *Geochimica et Cosmochimica Acta*, has isolated *Geobacteraceae* bacteria that turn soluble uranium found in groundwater into an insoluble form. This solidified form clings to geological formations underground and is thus immobilized. But questions remain, says Kostka: “Is the uranium stable? Is it going to remain in the ground? Are natural forces such as rainfall going to affect the stability of the uranium?” Kostka and his colleagues are currently working to answer these questions.

Another aspect of Kostka’s work involves providing a carbon source—food—for the bacteria. The research described in *Geochimica et Cosmochimica Acta* evaluates two forms of carbon, ethanol and glucose. “Ethanol is easier to handle on a large scale [although] glucose has also been shown to be effective,” he says. These compounds are “fed” to the bacteria through injection wells.

Microbiologist Colleen Hansel of Harvard University says, “Very little is known about these bacteria and the process that is involved in uranium immobilization in sediments and waters, so starting to understand the bacteria and genetically how these organisms do this process is very important. There is still so much we don’t know.” For example, it is important to learn how to best deliver nutrients to the bacteria. And it’s important to pinpoint which bacteria to foster—scientists want to stimulate the growth of those that will immobilize uranium and not others that might interfere with that process.

Still, the idea of using bacteria to treat radioactive waste may have broad and important implications, says Kostka, since DOE facilities that once made nuclear weapons have vast contaminated underground areas. “I think immobilizing [uranium waste] by microbial techniques is a good strategy, because the necessary bacteria are present in the environment already,” says Andreas Kappler, a geomicrobiologist at the University of Tübingen, Germany.

